# Spatial Frequency Responses of Anisotropic Refractive Index Gratings Formed in Holographic Polymer Dispersed Liquid Crystals

**DOI:** 10.3390/ma9030188

**Published:** 2016-03-10

**Authors:** Yoshiaki Fukuda, Yasuo Tomita

**Affiliations:** Department of Engineering Science, University of Electro-Communications, 1-5-1 Chofugaoka, Chofu, Tokyo 182-8585, Japan; yoshiaki.fukuda@jp.sony.com

**Keywords:** holographic polymer dispersed liquid crystal, photopolymer, nematic liquid crystal, phase separation, holographic grating formation, Bragg grating

## Abstract

We report on an experimental investigation of spatial frequency responses of anisotropic transmission refractive index gratings formed in holographic polymer dispersed liquid crystals (HPDLCs). We studied two different types of HPDLC materials employing two different monomer systems: one with acrylate monomer capable of radical mediated chain-growth polymerizations and the other with thiol-ene monomer capable of step-growth polymerizations. It was found that the photopolymerization kinetics of the two HPDLC materials could be well explained by the autocatalytic model. We also measured grating-spacing dependences of anisotropic refractive index gratings at a recording wavelength of 532 nm. It was found that the HPDLC material with the thiol-ene monomer gave higher spatial frequency responses than that with the acrylate monomer. Statistical thermodynamic simulation suggested that such a spatial frequency dependence was attributed primarily to a difference in the size of formed liquid crystal droplets due to different photopolymerization mechanisms.

## 1. Introduction

Liquid crystals (LCs) have been extensively studied because of their extremely large electrooptic responses that are useful for many photonic applications. These applications include various types of LC displays [1], tunable LC devices [2], and LC lasers [3]. Moreover, LC-polymer composites known as holographic polymer dispersed liquid crystals (HPDLCs) have also been investigated so far [4,5,6]. Such electro-optic HPDLCs consist of LCs incorporated into photopolymer followed by holographic polymerization that represents a fast and relatively simple way of fabricating multi-dimensional refractive index grating structures for electrically switchable and tunable photonic devices [7]. They have been used for wavelength filters, 3D displays, hyperspectral imaging, optical beam switching/control devices, sensors, lasers, optical memory and photonic crystals [8,9,10,11,12,13,14,15,16,17,18,19,20,21,22,23,24].

For the realization of efficient HPDLC photonic devices it is necessary to characterize spatial frequency responses of formed HPDLC gratings as many applications require large refractive index modulation amplitudes Δn at spatial frequencies of the order of 1000 lines/mm (*i.e.*, grating spacing $\Lambda_{g}$ = 1 μm or shorter). It is well known that Δn formed in binder-based photopolymer has a strong dependence on spatial frequency (or $\Lambda_{g}$) as a result of the photopolymerization-driven reaction-diffusion process [25] and a nonlocal response effect due to the finite chain length of growing polymer [26] in the holographic recording process. Although the former process usually causes a reduction in Δn at low spatial frequencies ($\Lambda_{g}>$ 1 μm), the latter effect suppresses Δn at high spatial frequencies ($\Lambda_{g}<$ 1 μm). Therefore, Δn formed in binder-based photopolymer is maximized more or less near $\Lambda_{g}$ = 1 μm [27]. On the other hand, multi-component photopolymer materials incorporating photo-insensitive species such as polymer liquid crystal polymer slices (POLICRYPS) [28,29], a variant of HPLDCs without the LC droplet formation, and nanoparticle-polymer composites (NPCs) [30,31] also possess a characteristic spatial frequency dependence due to their mass transport mechanism during holographic grating formation. Such a dependence observed in POLICRYPS was theoretically studied by a phenomenological reaction-diffusion model in terms of a concept of passive volume of growing polymers [28,32]. To the best of our knowledge, however, no systematic study on the spatial frequency response of HPDLC gratings accompanied with the LC droplet formation [5,33] has been reported so far.

In order to understand the holographic grating formation and the grating characteristics (including its spatial frequency dependence) in HPDLCs, one needs an appropriate theoretical model that can explain physico-chemical phenomena involving the polymerization-driven reaction-diffusion kinetics of monomer, polymer and LC followed by phase separation and LC nematic ordering in an HPDLC under holographic exposure. Various theoretical models have been proposed so far, which are classified into two types: a phenomenological reaction-diffusion model (model 1) [28,32,34,35,36,37,38] and a statistical thermodynamic model (model 2) [39,40,41,42,43]. The model 1 describes the photopolymerization-driven reaction-diffusion kinetics of the monomer-LC mixture in the isotropic mixing phase, but the kinetics of phase separation and LC nematic ordering during holographic polymerization are not naturally included. Therefore, the anisotropic nature of Δn as a result of LC nematic ordering in formed LC droplets cannot be evaluated. This drawback was overcome by Sutherland *et al.* [38] who proposed a phenomenological model that combined the reaction-diffusion model with the Maier-Saupe theory of LC nematic ordering [44] to account for the induced optical anisotropy of Δn in the transient and steady states. On the other hand, the model 2 directly treats the phase separation and LC nematic ordering leading to the formation of anisotropic grating structures. Kyu *et al.* performed numerical simulations of the dynamics of LC morphology and nematic ordering during photopolymerization-induced phase separation in an HPDLC film [39,40,41,42,43]. They used a two-dimensional coarse-grained continuous field model based on a time-dependent Ginzburg-Landau (TDGL) theory to calculate the spatio-temporal evolution of LC density and nematic ordering distributions during the photopolymerization-induced phase separation. Because they assumed emerging polymer as being completely miscible with the residual monomer (*i.e.*, the homogeneous mixture of emerging polymer with the residual monomer), the system could be regarded as a pseudo-two-component system in the free energy calculation. They also assumed the first-order polymerization reaction for simplicity. Later, we extended their analysis by considering a generic three-component system consisting of monomer, polymer and LC together with a more realistic autocatalytic reaction that could explain better the polymerization kinetics of acrylate monomer in HPDLCs [45].

In this paper we describe experiments to study spatial frequency responses of anisotropic transmission gratings formed in HPDLCs using two different types of monomers: one with acrylates showing the radical mediated chain-growth polymerizations and the other with thiol-ene monomers showing radical mediated step-growth polymerizations [46]. We measure grating-spacing dependences of anisotropic refractive index gratings formed in these two types of HPDLC materials for *p* and *s* polarized readout. We discuss measured results in reference to numerical simulation results using our statistical thermodynamic model [45] that is briefly described in Appendix A here.

## 2. Experimental Results and Discussion

### 2.1. Materials

**Figure 1:**
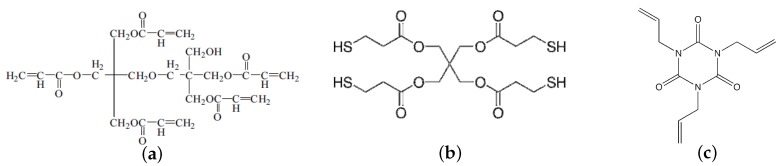

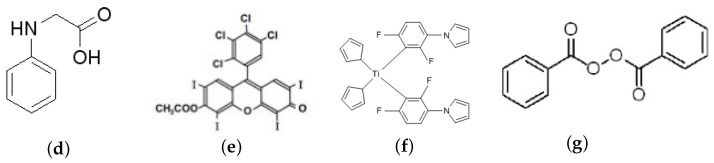
Chemical structures of (**a**) DPEPHA; (**b**) tetrathiol; (**c**) TATATO; (**d**) NPG; (**e**) RB; (**f**) Irgacure 784; and (**g**) BzO${}_{2}$.

We employed two different types of HPDLC materials: sample I used acrylate monomer capable of radical mediated chain-growth polymerizations, which is a typical HPDLC formulation, and sample II used thiol-ene monomer capable of radical mediated thiol-ene polymerizations. In these HPDLC materials nematic LC (E7, Merck) having extraordinary and ordinary refractive indices of $n_{e}$ = 1.7305 and $n_{o}$ = 1.5189, respectively and the nematic-isotropic transition temperature $T_{NI}$ of 60 °C were used. Specifically, sample I consists of 30 vol.% E7 and multifunctional acrylate monomer, dipentaerithrytol penta-hexa acrylate (DPEPHA, Aldrich), together with 1 wt.% *N*-phenyl-glycine (NPG, Tokyo Chemical Industry) and 1 wt.% Rose Bengal (RB, Tokyo Chemical Industry) used for green light sensitization. Sample II consists of 30 vol.% E7 and a stoichiometric composition of a pentaerythritol tetrakis(3-mercaptopropionate) (tetrathiol, Aldrich) and an allyl triazine triene monomer, triallyl-1,3,5-triazine-2,4,6(1H,3H,5H)-trione (TATATO, Aldrich), together with a green light initiator system of 2 wt.% Irgacure 784 and 2.5 wt.% BzO${}_{2}$. Since the average functionality of this thiol-ene combination is greater than two, a crosslink thiol-ene polymer network can be formed [47]. Furthermore, TATATO possesses the rigid structure of the triazine group, the high electron density of the double bond and homopolymerization characteristics, giving fast thiol-ene polymerization rates, moderately late gel point conversion and increased cross-linking network densities [48,49]. Note that 30 vol.% of E7 was used since it gave the largest values for saturated refractive index modulation amplitudes $\Delta n_{\mathrm{sat}}$. These chemical formulae are shown in Figure 1. Refractive indices of monomer and polymer ($n_{m}$, $n_{p}$) were (1.4866, 1.5213) for DPEPHA. Refractive indices of tetrathiol and TATATO were 1.5312 and 1.513, respectively, and the polymer refractive index of the stoichiometric tetrathiol and TATATO was 1.5477. In our holographic recording measurement the mixed syrup of each sample was cast on a glass plate and was covered with another 10-*μ*m-spacer loaded glass plate to make film samples. It is well known that thick (≫10 μm) HPDLC films experience strong holographic scattering during recording due to the formation of high contrast LC droplets [50]. In addition to such a detrimental effect for thick HPDLC films, it is preferable to maintain a film thickness thin, say, of the order of 10 μm since a high electric field with a relatively low bias voltage can be applied in a film. For these reasons film thickness of the order of 10 μm is substantive. In this case it is necessary to have $\Delta n_{\mathrm{sat}}$ closer to or larger than 0.01 to obtain high diffraction efficiencies. Note that no transparent electrode was coated on the glass substrates so that an external electric field was not applied between the glass substrates in our experiment. This is so because our primary purpose in this study is to investigate the effect of LC droplet formation on spatial frequency responses of HPDLC transmission gratings.

### 2.2. Photopolymerization Kinetics

Since the temporal growth and the steady-state morphology of LC density and orientation order parameters strongly depends on the polymerization kinetics, it is expected that the average size of LC droplets is also influenced by the polymerization kinetics. In order to examine a difference in LC droplet morphology between samples I and II, we first measured the photopolymerization kinetics. We employed a commercially available a photo-differential scanning calorimeter (Q200, TA instrument) equipped with a refrigerated cooling system (RCS 90, TA instrument) to accurately maintain the isotherm condition at 25 °C. The mixed syrup of each sample described in the previous subsection was dripped on an uncovered aluminum pan and the weight was approximately 5.5 mg. The sample chamber of the photocalorimeter was purged with nitrogen gas for 30 min prior to light irradiation to avoid oxygen inhibition. Photopolymerization was initiated by a loosely focused light beam from a 200 W Hg-Xe lump through a 532-nm bandpass filter and a light-guiding fiber bundle. The curing light intensity at the mixed syrup was set to be 50 mW/cm${}^{2}$ during the measurement. The conditions for the operating temperature and the recording intensity was the same as those in our holographic recording experiment and its reason is described in Section 2.3. The detailed procedure of measuring the time evolution of polymer conversion is described in our previous work [51].

In order to understand measured results, we employed the so-called autocatalytic model [52,53,54] that considers the formation of some intermediate species markedly accelerating the initial reaction. In this model the relative conversion $\alpha_{r}$, defined as the time-dependent polymer conversion normalized by its final value, has the following relation to the polymerization rate constant $d\alpha_{r}/dt$:

(1) $$\frac{d\alpha_{r}}{dt}=k\alpha_{r}^{m}{\left(1-\alpha_{r}\right)}^{n}$$

where the Arrhenius-type polymerization rate constant is given by *k* that may be approximated to $k_{0}I^{\gamma }$ in which $k_{0}$ is the coefficient of polymerization kinetics as a function of $\exp(-E_{a}/k_{B}T)$ ($E_{a}$ is the activation energy of reaction and $k_{B}T$ is the thermal energy); *I* is the absorbed light intensity; and the exponent *γ* depends on the polymerization kinetic process. Also, *m* is the reaction order exponent characterizing the growth of polymer chains and *n* is the autocatalytic exponent characterizing the rate of monomer consumed. We note that the first-order single reaction model employed by Meng *et al.* [40,43] and Nwabunma *et al.* [55,56] is given with m=0 and n=1. Under the approximation that all initiating radical and growing polymer radical concentrations quickly reach the steady state in the radical chain-growth process (*i.e.,* the quasi-steady state approximation) *γ* is 0.5 [46,55,57]. This assumption was used to describe the holographic grating formation in photopolymers and HPDLCs in several phenomenological [28,34,35,36,37,38] and statistical thermodynamic [41] models, while the assumption of γ=1 was also considered previously [39,40,41,42,43,55,58,59]. In our case the former value was used since it gave good agreement with measured data as shown below.

**Figure 2:**
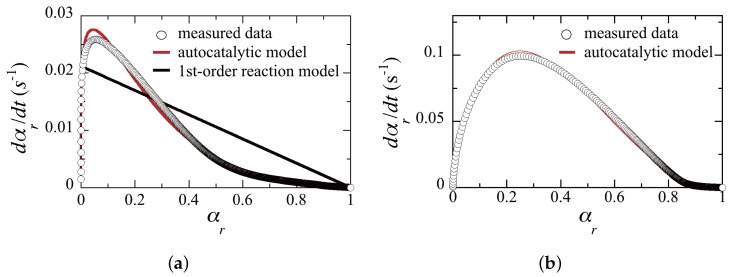
Relative conversion $\alpha_{r}$ versus $d\alpha_{r}/dt$ for (**a**) sample I; and (**b**) sample II at a curing wavelength of 532 nm and at 25 °C.

Figure 2a shows measured data (open circles) of $\alpha_{r}$ *vs*. $d\alpha_{r}/dt$ for sample I. The solid curves correspond to the least-squares fits of Equation (1) for the autocatalytic model (the curve in red) with varied *m* and *n* and for the first-order single reaction model (the curve in black) with m=0 and n=1 to the measured data. It can be seen that the autocatalytic model with k=0.045 s${}^{-1}$ ($k_{0}=0.20$ cm/s·W${}^{0.5}$), m=0.14 and n=2.86 is in good agreement with the measured data, while the first-order single reaction model with k=0.022 s${}^{-1}$ shows a very poor fit. This result indicates that the autocatalytic model is a good approximation to describe the radical mediated chain-growth polymerization process. It can also be seen that the gelation point (the conversion point where $d\alpha_{r}/dt$ is maximized) occurs at $\alpha_{r}\approx 0.05$ (*i.e.*, 5% polymer conversion), typical for the chain-growth reaction that exhibits rapid gelation at very early conversion. Figure 2b shows measured data (open circles) for sample II, where the solid curve corresponds to the least-squares fits of Equation (1) for the autocatalytic model (the curve in red) with varied *m* and *n* to the measured data. No curve fit is shown for the first-order single reaction model since it does not obviously fit the measured data. It can be seen that the autocatalytic model with k=0.54 s${}^{-1}$ ($k_{0}=2.40$ cm/s·W${}^{0.5}$), m=0.71 and n=2.24 is in good agreement with the measured data. It can also be seen that the gelation point occurs at $\alpha_{r}\approx 0.22$, approximately four times larger than that for sample I. A trend of the late gelation is a typical characteristics of thiol-ene polymerizations and results in drastic shrinkage suppression of holographic volume gratings recorded in NPCs [60,61,62]. Also, $k_{0}$ for sample II is much larger than that for sample I, showing that the polymerization speed is also faster at a given *I* due to efficient thiol-ene reaction by use of TATATO. These results suggest that the size of an LC droplet is smaller for sample II than that for sample I since higher polymerization rates and delayed gelation make the droplet size smaller [63]. This point will be examined experimentally in the next subsection.

### 2.3. Holographic Recording

**Figure 3:**
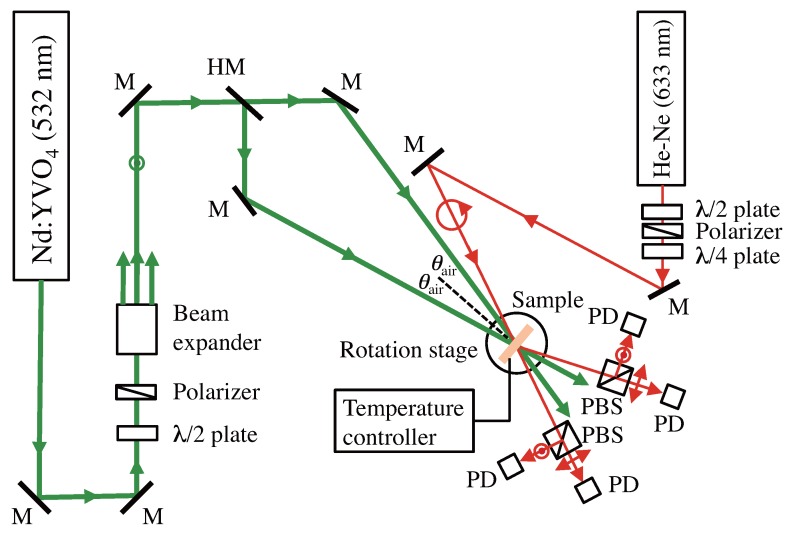
Experimental setup for holographic recording and measuring refractive index modulation amplitudes of anisotropic refractive index gratings. M: mirror; HM: half mirror; PD: photodetector; PBS: polarizing beam splitter.

It is well known that a recorded holographic grating in an HPDLC film is highly anisotropic due to the nematic ordering of LC molecules within LC droplets formed in the dark regions of intensity-interference fringe pattern [38]. Therefore, we constructed an optical setup for recording and reading out anisotropic holographic gratings being formed in our samples at a recording wavelength of 532 nm, as shown in Figure 3. We employed a linearly polarized laser beam from a frequency-doubled diode-pumped Nd:YVO${}_{4}$ laser operating at 532 nm as a coherent light source. After passing through a tandem combination of a half-wave plate, a polarizer and a beam expander, the collimated and expanded beam was *s*-polarized and was divided into two beams of equal intensities at an incident half angle $\theta_{air}$ to record a unslanted plane-wave holographic grating in a film sample that was placed on a rotation stage and the sample temperature was kept at 25 °C by a temperature controller. This ambient temperature setting stems from our motivation, that is, an investigation of how the spatial frequency response of an HPDLC grating, unlike a POLICRYPS grating formed by high temperature recording above $T_{NI}$ [29], is influenced by the LC droplet formation. In our experiment the total recording intensity was kept at 50 mW/cm${}^{2}$ that gave the maximum $\Delta n_{\mathrm{sat}}$ at $\Lambda_{g}$ = 1 *μ*m. In order to measure the time evolution of anisotropic diffraction efficiencies from an HPDLC grating, we used a Bragg-matched circularly polarized readout beam derived from a linearly polarized He-Ne laser at a photo-insensitive wavelength of 633 nm through polarizing components. Mutually orthogonal *p*-polarized (in the diffraction plane) and *s*-polarized (perpendicular to the diffraction plane) components of transmitted and diffracted beams from a film sample being recorded were spatially separated by polarizing beam splitters after the sample to calculate diffraction efficiencies $\eta_{p}$ and $\eta_{s}$ at *p* and *s* polarizations, respectively, where $\eta_{p}$ ($\eta_{s}$) was defined as the ratio of the 1st-order diffracted signal to the sum of the 0th- and 1st-order signals at *p* (*s*) polarization. We note that although we did not employ an active fringe-stabilization system that could avoid the possible formation of slanted and/or bended gratings due to phase fluctuations of two recording beams [64,65], no such a negative effect was found in our experiment since no Bragg-angle change of a recorded grating was measured for samples I and II. The effective thickness *ℓ* of a film sample was estimated from a least-squares curve fit to the Bragg-angle detuning data of the saturated $\eta_{s}$ with Kogelnik’s formula for an unslanted transmission grating for *s* polarized readout [66]. Then, anisotropic values for $\Delta n_{\mathrm{sat}}$ at *p* and *s* polarizations, $\Delta n_{\mathrm{sat}}^{\left(p\right)}$ and $\Delta n_{\mathrm{sat}}^{\left(s\right)}$, were extracted from measured values for saturated $\eta_{p}$ and $\eta_{s}$ with *ℓ* and Kogelnik’s formulae for $\eta_{p}$ and $\eta_{s}$ given by:

(2a) $$\begin{array}{cc} \eta_{p} & =\sin^{2}\left[\frac{\pi \Delta n_{\mathrm{sat}}^{\left(p\right)}\ell \cos\left(2\theta_{B}\right)}{\lambda \cos\theta_{B}}\right] \end{array}$$

(2b) $$\begin{array}{cc} \eta_{s} & =\sin^{2}\left[\frac{\pi \Delta n_{\mathrm{sat}}^{\left(s\right)}\ell }{\lambda \cos\theta_{B}}\right] \end{array}$$

at *p* and *s* polarizations, respectively; where *λ* is a readout wavelength in vacuum; and $\theta_{B}$ is the Bragg angle inside a film sample.

**Figure 4:**
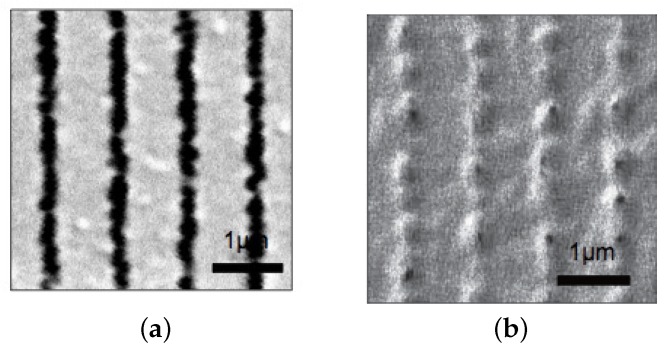
SEM images of recorded HPDLC gratings at $\Lambda_{g}$ = 1 μm for (**a**) sample I; and (**b**) sample II. Dark portions correspond to LC-rich regions.

Figure 4 shows scanning electron microscope (SEM) images of morphologies of plane-wave gratings recorded at $\Lambda_{g}=1$ μm for sample I (Figure 4a) and sample II (Figure 4b). Note that the grating vector of the intensity-interference fringe pattern is along the horizontal direction. These samples were prepared by dipping recorded HPDLC film samples into methanol and the polymer structures along the cross sectional plane parallel to a glass substrate (also to the grating vector) were observed by SEM. It is observed that while formed LC droplets were elongated along the equi-intensity direction (perpendicular to the grating vector) for sample I, they are smaller and less elongated for sample II as compared to those of sample I. A similar configuration of LC droplets embedded in an HPDLC transmission grating was reported by Jazbinšek *et al.* [67] although the formulation of their acrylate-based HPDLC formulation was different from ours. They reported that the director direction of LC molecules within an elongated LC droplet was along the minor droplet axis. In our case it can be examined by measuring the anisotropic diffraction properties of formed HPDLC gratings, which will be described later.

**Figure 5:**
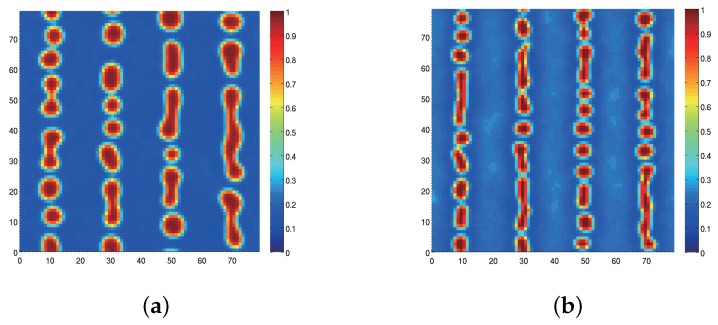
Calculated steady-state morphologies of the LC density order parameters for (**a**) sample I; and (**b**) sample II under two plane-wave holographic exposure at $\Lambda_{g}$ = 1 *μ*m. The initial LC density is 30 vol.%.

Figure 5 shows calculated results of steady-state spatial distributions of the LC density order parameters at $\Lambda_{g}=$ 1 *μ*m for sample I (Figure 5a) and sample II (Figure 5b) by means of our numerical simulation [45] outlined in Appendix A. The total recording intensity of 50 mW/cm${}^{2}$ and the sample temperature of 25 °C were used in the calculation. It can be seen that the calculated morphologies are generally similar to the measured ones shown in Figure 4: the calculated average size of LC droplets was approximately 200 nm and 147 nm for samples I and II, respectively, in consistent with our observation shown in Figure 4. It is known that the two-phase morphologies between these two different monomer types in an HPDLC transmission grating differ each other: acrylate-based HPDLCs such as sample I possess inhomogeneous LC droplet size and non-spherical shape, while a thiol-ene-based HPDLC such as sample II has homogeneous droplet size and spherical shape [68,69,70]. Such a qualitative difference may be explained by the fact that while long chain polymer is formed immediately in the chain-growth polymerization (see Figure 2a), relatively slow gelation occurs in the step-growth polymerization process (see Figure 2b). Namely, in the former case the gelation precedes phase separation and the polymer-rich discontinuous phase of long chain polymer continues to grow, forming irregular (non-spherical) shaped large LC droplet domains [69]. In the latter case, however, the phase separation precedes gelation and phase separation of an oligomer-rich liquid from an LC-rich liquid takes place, forming LC droplets with spherical shape in low viscosity liquid as a result of surface tension effects [69]. Because of such a morphological difference thiol-ene-based HPDLCs are sometimes favored to obtain better optical and electrical properties and long-term stability in the UV and visible wavelength regions [69,70]. The results shown in Figure 4 and Figure 5 suggest that an HPDLC transmission grating with sample II based on thiol-ene polymerizations possess a better spatial frequency response than that with sample I since LC droplets in sample II are smaller than those in sample I.

**Figure 6:**
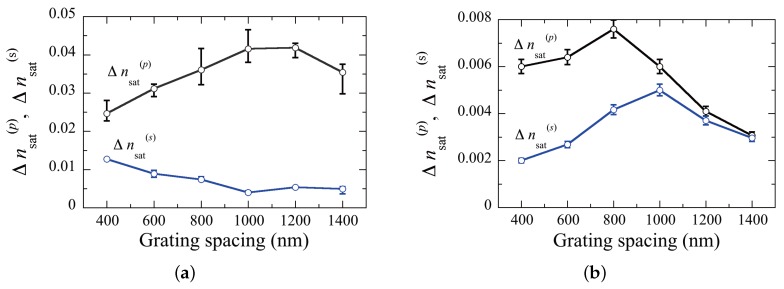
Measured grating-spacing dependences of $\Delta n_{\mathrm{sat}}^{\left(p\right)}$ and $\Delta n_{\mathrm{sat}}^{\left(s\right)}$ for (**a**) sample I; and (**b**) sample II.

Figure 6 shows measured grating-spacing dependences of $\Delta n_{\mathrm{sat}}^{\left(p\right)}$ and $\Delta n_{\mathrm{sat}}^{\left(s\right)}$ for samples I and II. It can be seen that $\Delta n_{\mathrm{sat}}^{\left(p\right)}$ is larger than $\Delta n_{\mathrm{sat}}^{\left(s\right)}$ at all measured $\Lambda_{g}$s. This result implies that the director direction of LC molecules within an elongated LC droplet was along the minor droplet axis. It can also be seen that, as observed in POLICRYPS [28] and NPCs [30,31], there exist the optimum values for $\Lambda_{g}$ maximizing $\Delta n_{\mathrm{sat}}^{\left(p\right)}$ for samples I and II. A similar trend in $\Delta n_{\mathrm{sat}}^{\left(s\right)}$ is seen for sample II (Figure 6b) but not for sample I (Figure 6a). We speculate that this discrepancy is attributed to the director direction of LC molecules within a relatively large LC droplet in sample I: as $\Lambda_{g}$ becomes comparable to the size of LC droplets, LC droplets tend to randomly distribute with their less elongated shape. In this case the director of LC molecules within such a less elongated LC droplet tends to have less preferred direction, so that $\Delta n_{\mathrm{sat}}^{\left(s\right)}$ increases with decreasing $\Lambda_{g}$ while $\Delta n_{\mathrm{sat}}^{\left(p\right)}$ decreases. It can also be seen that values for $\Delta n_{\mathrm{sat}}^{\left(p\right)}$ are lower for sample II than those for sample I. This difference is attributed to the optical anisotropy of formed LC droplets and to the refractive index difference between optically anisotropic LC and formed polymers for samples I and II.

**Figure 7:**
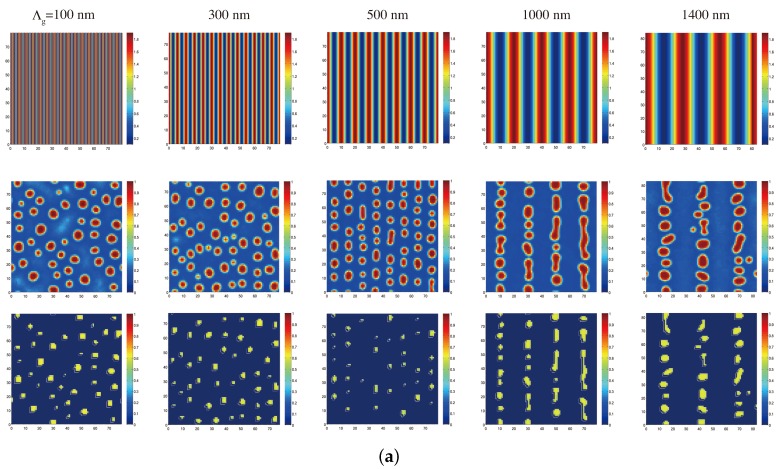

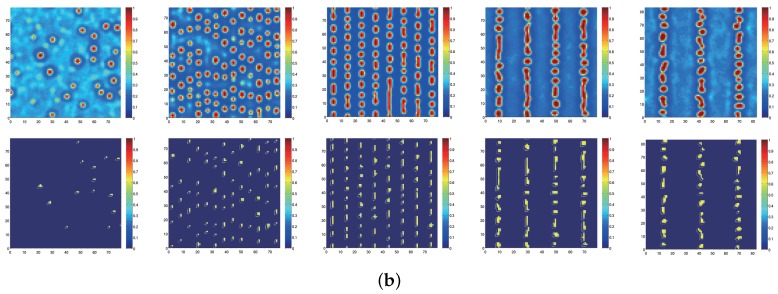
Calculated steady-state morphologies of the LC density (the second row) and orientation (the third row) order parameters for (**a**) sample I; and (**b**) sample II under two plane-wave holographic exposure at $\Lambda_{g}$ of 100, 300, 500, 1000 and 1400 nm. The sinusoidal intensity-interference pattern (first row) is also shown. The initial LC density is 30 vol.%.

In order to examine a morphological dependence of LC droplets on $\Lambda_{g}$, we performed numerical simulations as shown in Figure 7. It illustrates calculated results of the steady-state morphologies of the LC density and orientation order parameters for sample I (Figure 7a) and sample II (Figure 7b) under two plane-wave holographic exposure at different values for $\Lambda_{g}$. It can be seen that LC droplets are formed and assembled in the dark regions of the intensity-interference fringe pattern at longer values for $\Lambda_{g}$. This is caused by the photopolymerization-driven mutual diffusion of monomer and LC molecules as also found between monomer and nanoparticles in NPC gratings [71,72]. However, such periodic assembly tends to disappear at shorter $\Lambda_{g}$ (<300 nm). It can be seen that LC droplets at $\Lambda_{g}$ = 100 nm are randomly distributed, as similar to that of PDLCs under uniform curing [68]. Such a trend becomes significant as $\Lambda_{g}$ is closer to and shorter than the droplet size. For example, while the periodic distribution of LC droplets is seen in sample II at $\Lambda_{g}$ = 300 nm, it is barely seen in sample I. These features reflect on a difference in the average size of LC droplets between samples I and II as seen in Figure 4 and Figure 5. Such a trend results in the high spatial frequency cut off of the induced refractive index grating. For this reason $\Delta n_{\mathrm{sat}}^{\left(p\right)}$ is peaked at longer $\Lambda_{g}$ for sample I than for sample II since the average size of LC droplets in sample I is larger than that in sample II. LC droplets strongly “interact" one another during their formation when $\Lambda_{g}$ is comparable to the droplet size that is determined by the polymerization and phase separation kinetics. On the other hand, as $\Lambda_{g}$ is longer than 1000 nm, LC droplets tend to appear in the region between adjacent dark regions due to incomplete phase separation. In this case they are not positioned along the equi-intensity position, changing the shape of LC droplets from non-spherical to spherical structures as reported previously [67]. Such a trend results in a decrease in $\Delta n_{\mathrm{sat}}^{\left(p\right)}$ and $\Delta n_{\mathrm{sat}}^{\left(s\right)}$. It can be seen that the distribution of the LC orientation order parameters follow a similar dependence to the LC density order parameters. To describe the orientation of the LC director more rigorously, it is necessary to extend the present two-dimensional model to a three-dimensional model in which the LC ’s orientation order parameter should be treated as tensor [73]. We note that the result obtained here is completely different from Kyu *et al.*’s finding in which the preferential growth of LC droplets along the equi-intensity direction is seen with a decrease in $\Lambda_{g}$ because of their high concentration of LC (75 vol.%) [39].

## 3. Conclusions

We have experimentally studied spatial frequency responses of anisotropic transmission gratings formed in two types of HPDLC materials using monomers capable of radical mediated chain-growth (acrylate) and step-growth (thiol-ene) photopolymerizations. We have seen that HPDLC gratings using acrylate monomer generally contain large size of LC droplets and thereby $\Delta n_{\mathrm{sat}}^{\left(p\right)}$ tend to decrease rapidly at high spatial frequencies. This trend is caused by rapid gelation at very early conversion. On the other hand, HPDLC gratings using thiol-ene monomer contain smaller size of LC droplets, giving better spatial frequency response at high spatial frequencies. This property is attributed to late gelation in step-growth polymerizations as compared to the case of chain-growth polymerizations. These results indicate that HPDLC gratings using low-shrinkage thiol-ene monomers are suitable for applications such as lasers, tunable filters and sensors, where HPLDC reflection gratings at short $\Lambda_{g}$s are usually employed. We have also compared measured results with those of the numerical simulation. We have shown that the numerical simulation provides good qualitative explanations for measured results on spatial frequency responses of HPDLC gratings. For quantitative discussions more elaborated theories such as a three-dimensional model including the tensor treatment of the LC orientation order parameter may be necessary to understand interesting soft matter physical phenomena occurring in HPDLCs under holographic polymerization and to obtain better design methodologies of HPDLC devices. Furthermore, an investigation of the spatial frequency dependent electro-optic properties of HPDLC gratings is also of great importance for device applications. Our efforts along these directions are underway.

## Appendix A : Theoretical Formulation for Statistical Thermodynamic Simulations

Various continuous field theories have been reported to study the formation of mesoscopic micellar structures in soft matter physics. For example, the dynamic computer simulation using the self-consistent field theory provides accurate results for both weak and strong segregation for this purpose, but it requires large CPU power and memory spaces and therefore it is rather computational intensive. On the other hand, the TDGL simulation requires less CPU power, enabling one to perform large scale numerical simulations. However, the TDGL calculation is usually limited to weak segregation due to the fact that the free energy of the system is expanded to a few low orders in a series of order parameter fluctuations (*i.e.*, the Ginzburg-Landau expansion) [74]. Holographic photopolymerization in HPDLCs being discussed here, however, induces the phase separation accompanied with strong segregation of LCs. Therefore, Meng *et al.*’s theoretical treatment [40,43], the model 2, may be suited for numerical simulations in the strong segregation regime with less computational cost since the free energy is expressed as a functional of density fields of constituents. In what follows we briefly describe our statistical thermodynamic analysis reported in Ref. [45] that applies the model 2.

The reaction-diffusion kinetics of the photopolymerization-induced pahse separation is modeled in terms of a conserved density order parameter $\phi_{i}\left(r,t\right)$ for LC (i=L), monomer (i=m) and polymer (i=p) at a position r and a time *t* as well as of a nonconserved orientation order parameter s(r,t) of the LC at the same position and time. It is generally given by [39,42]:

(A1a) $$\begin{array}{cc} \frac{\partial \phi_{L}}{\partial t} & =\nabla \cdot \left[\Lambda \nabla \left(\frac{\delta G}{\delta \phi_{L}}\right)\right]+\eta_{\phi_{L}} \end{array}$$

(A1b) $$\begin{array}{cc} \frac{\partial \phi_{m}}{\partial t} & =\nabla \cdot \left[\Lambda \nabla \left(\frac{\delta G}{\delta \phi_{m}}\right)\right]-u+\eta_{\phi_{m}} \end{array}$$

(A1c) $$\begin{array}{cc} \frac{\partial \phi_{p}}{\partial t} & =\nabla \cdot \left[\Lambda \nabla \left(\frac{\delta G}{\delta \phi_{p}}\right)\right]+u+\eta_{\phi_{p}} \end{array}$$

(A1d) $$\begin{array}{cc} \frac{\partial s}{\partial t} & =-R_{s}\left(\frac{\delta G}{\delta s}\right)+\eta_{s} \end{array}$$

where Λ is the mutual diffusion coefficient; *G* is the total free energy of the system; *u* is the photopolymerization rate; and $\eta_{i}$ is the Langevin noise for the density and orientation fields ($i=\;\phi_{L},\phi_{m},\phi_{p},s$) with the following constraint due to the fluctuation-dissipation theorem [56]:

(A2a) $$\begin{array}{cc} <\eta_{\phi_{i}}\left(r,t\right)\eta_{\phi_{j}}\left(r^{\prime },t^{\prime }\right)> & =-2k_{B}T\Lambda \nabla^{2}\delta \left(r-r^{\prime }\right)\delta \left(t-t^{\prime }\right)\delta_{ij} \end{array}$$

(A2b) $$\begin{array}{cc} <\eta_{s}\left(r,t\right)\eta_{s}\left(r^{\prime },t^{\prime }\right)> & =-2k_{B}TR_{s}\delta \left(r-r^{\prime }\right)\delta \left(t-t^{\prime }\right) \end{array}$$

in which $k_{B}T$ is the thermal energy; and $R_{s}$ is a coefficient related to the rotational mobility of the LC director, respectively.

The mutual diffusion coefficient Λ in Equation (A1) has the Onsager reciprocity relation [75] given by:

(A3) $$\begin{array}{c} \Lambda =\frac{\Lambda_{L}\Lambda_{m}+\Lambda_{L}\Lambda_{p}+\Lambda_{m}\Lambda_{p}}{\Lambda_{L}+\Lambda_{m}+\Lambda_{p}} \end{array}$$

where $\Lambda_{i}$ is the diffusion coefficient of the *i*th component (i=L,m,p) as given by

(A4) $$\begin{array}{cc} \Lambda_{i} & =\phi_{i}r_{i}D_{i} \end{array}$$

in which $r_{i}$ and $D_{i}$ are the statistical chain segment length and the self-diffusion constant of the *i*th component, respectively. Since the sizes of LC and monomer are more or less similar, it may be legitimate to set $r_{L}$ and $r_{m}$ to be unity for simplicity [39,40,41,43,56]. In addition, $r_{p}$ is given by [59]:

(A5) $$\begin{array}{c} r_{p}=\frac{1}{1-f\alpha /2} \end{array}$$

where f(≥2) is the functionality of multifunctional monomer (necessary to induce the crosslinking network structure for the efficient mutual diffusion of LC and monomer molecules under holographic exposure) and *α* is the polymer conversion given by [59]:

(A6) $$\begin{array}{c} \alpha =\frac{2(1-\phi_{m})}{f} \end{array}$$

In Equation (A4) $D_{i}$ is expressed, according to the reptation theory [76], as

(A7) $$\begin{array}{c} D_{i}=\frac{k_{B}T}{\zeta_{i}}\frac{r_{e}}{r_{i}^{2}} \end{array}$$

where $\zeta_{i}$ is the frictional coefficient of the *i*th component; and $r_{e}$ is the segment number between two successive entanglements of polymer chains. It should be noted that $r_{p}$ is usually taken very large ($r_{p}\to \infty$) so as to be $D_{p}=0$ and $\Lambda_{p}=0$ in the past works since the emerging polymer chains are fixed at the chemical junction due to the charactristic of the cross-linking reaction [39,40,41,42,43,56]. It means that the diffusion term of emerging polymers on the right-hand side of Equation (A1c) is neglected. However, we shall keep $r_{p}$ finite to take the possible diffusion of early growing polymer radicals (e.g., oligomers) into account.

We assume that the rate at which monomers disappear, $-d\phi_{m}/dt$, approximately equals to *u* since it is usually much larger than the initiation rate. Then, *u* may be rewritten, in terms of the autocatalytic model given by Equation (2) with γ=0.5, as

(A8) $$\begin{array}{cc} u & =k_{0}I^{0.5}\phi_{m}^{n}{\left(1-\phi_{m}\right)}^{m} \end{array}$$

The total free energy *G* of the mixture of LCs, monomer and polymer is given by:

(A9) $$\begin{array}{c} G=\int dr^{3}\left(g_{FH}+g_{n}+g_{e}+g_{r}+\sum_{i=L,m,p}\kappa_{\phi_{i}}|\nabla \phi_{i}{|}^{2}+\kappa_{s}{\left|\nabla s\right|}^{2}\right) \end{array}$$

where $g_{i}$ is the local free energy density of three-component isotropic mixing described by Flory-Huggins theory [77] as given by:

(A10) $$\begin{array}{c} g_{FH}=\frac{\phi_{L}\ln\phi_{L}}{r_{L}}+\frac{\phi_{m}\ln\phi_{m}}{r_{m}}+\frac{\phi_{p}\ln\phi_{p}}{r_{p}}+\chi_{L,m}\phi_{L}\phi_{m}+\chi_{L,p}\phi_{L}\phi_{p}+\chi_{m,p}\phi_{m}\phi_{p} \end{array}$$

in which $\chi_{ij}$ is the Flory-Huggins interaction parameters between the *i* and the *j* components, which takes a functional form of *A*+*B*/*T* (*A* and *B* being constants representing athermal entropic and enthalpic contributiond, respectively, and *T* being the system’s temperature) [59]. Also, $g_{n}$ is the local free energy density of LC nematic ordering described by the Maier-Saupe theory of nematic ordering [44] such that:

(A11) $$\begin{array}{c} g_{n}=\frac{1}{r_{L}}\left(-\phi_{L}\ln z+\frac{1}{2}\nu \phi_{L}^{2}s^{2}\right) \end{array}$$

where *ν* is the nematic interaction parameter given by $\nu =4.541T_{NI}/T$ and *z* is the partition function expressed as:

(A12) $$\begin{array}{c} z=\int_{0}^{1}\exp\left[\frac{\nu \phi_{L}s}{2}\left(3x^{2}-1\right)\right]dx \end{array}$$

in which *s* is given by:

(A13) $$\begin{array}{c} s=\frac{\int_{0}^{1}\frac{1}{2}\left(3x^{2}-1\right)\exp\left[\frac{\nu \phi_{L}s}{2}\left(3x^{2}-1\right)\right]dx}{z} \end{array}$$

The local elastic free energy density $g_{e}$ for a flexible crosslinked polymer chain obeying ideal Gaussian chain statistics [39,56] is given by:

(A14) $$\begin{array}{c} g_{e}=\frac{3\alpha_{e}}{2r_{c}}\Phi_{0}^{2/3}\left(\phi_{p}^{1/3}-\phi_{p}\right)+\frac{\beta_{e}}{r_{c}}\phi_{p}\ln\phi_{p} \end{array}$$

where $\alpha_{e}$ and $\beta_{e}$ are the network model parameters given by $\alpha_{e}=1$ and $\beta_{e}=2/f$ in the Flory’s affine network model [56] and $r_{c}$ is the segment length between crosslinked points being expressed, in terms of *α*, as [41,56]

(A15) $$\begin{array}{c} r_{c}=\frac{\alpha }{2-\alpha -2\sqrt{1-\alpha }} \end{array}$$

Note that $r_{c}$ is large when *α* is low but it decreases with an increase in *α*, indicating an increase in the crosslink density of the polymer network as the polymerization develops. In Equation (A14) the parameter $\Phi_{0}$ represents the reference volume fraction of the polymer network, which is identified as $\Phi_{0}=\phi_{p}$ (*i.e.,* the volume fraction of the polymer at the onset of crosslinking) [39] here.

The total mass conservation condition and the incompressibility condition can be maintained by introducing the constraint term $g_{r}$ given by [74]:

(A16) $$\begin{array}{c} g_{r}=\sum_{i=L,m,p}\lambda_{i}\left[\phi_{i}\left(r,t\right)-\phi_{i}\left(r,0\right)\right]+\kappa \left(r\right)\sum_{i=L,m,p}\left[\phi_{i}\left(r,t\right)-\phi_{i}\left(r,0\right)\right] \end{array}$$

where $\lambda_{i}$ and κ(r) are Lagrange multipliers corresponding to the total mass conservation condition and the incompressibility condition, respectively.

The fifth and sixth terms on the right-hand side of Equation (11) represent nonlocal free energy density terms associated with the gradients of the LC density and *s*, respectively, in which the interface gradient coefficient $\kappa_{\phi }$ for *i*th component is given by [59]:

(A17) $$\begin{array}{c} \kappa_{\phi_{i}}=\frac{1}{36}\frac{a_{i}^{2}}{\phi_{i}} \end{array}$$

where $a_{i}$ is the characteristic segment length of the *i*th component, which can be taken as unity, and the interface gradient coefficient $\kappa_{s}$ for LC orientation is usually taken as a constant for simplicity [39].
